# Novel *VPS13A* Gene Mutations Identified in Patients Diagnosed with Chorea-acanthocytosis (ChAc): Case Presentation and Literature Review

**DOI:** 10.3389/fnagi.2017.00095

**Published:** 2017-04-12

**Authors:** Yan Shen, Xiaoming Liu, Xi Long, Chao Han, Fang Wan, Wenliang Fan, Xingfang Guo, Kai Ma, Shiyi Guo, Luxi Wang, Yun Xia, Ling Liu, Jinsha Huang, Zhicheng Lin, Nian Xiong, Tao Wang

**Affiliations:** ^1^Department of Neurology, Union Hospital, Tongji Medical College, Huazhong University of Science and TechnologyWuhan, China; ^2^Department of Radiology, Union Hospital, Tongji Medical College, Huazhong University of Science and TechnologyWuhan, China; ^3^Department of Psychiatry, Division of Alcohol and Drug Abuse, and Mailman Neuroscience Research Center, McLean Hospital, Harvard Medical School, BelmontMA, USA

**Keywords:** chorea-acanthocytosis (ChAc), *VPS13A*, gene mutation, neuroimaging, caudate nucleus

## Abstract

Chorea-acanthocytosis (ChAc) is a rare autosomal recessive inherited syndrome characterized by hyperkinetic movements, seizures, cognitive impairment, neuropsychiatric symptoms, elevated serum biochemical indicators and acanthocytes detection in peripheral blood smear. Vacuolar protein sorting 13A *(VPS13A)* gene mutations have been proven to be genetically responsible for the pathogenesis of ChAc. Herein, based on the typical clinical symptoms and neuroimaging features, we present two suspected ChAc cases which are further genetically confirmed by four novel *VPS13A* gene mutations. Nevertheless, the sharp contrast between the population base and published ChAc reports implies that ChAc is considerably underdiagnosed in China. Therefore, we conclude several suggestive features and propose a diagnostic path of ChAc from a clinical, genetic and neuroimaging perspective, aiming to facilitate the diagnosis and management of ChAc in China.

## Introduction

Neuroacanthocytosis (NA) syndromes encompass a group of rare diseases characterized by the presence of “thorny” red blood cells (acanthocytes) in peripheral blood smear and neurodegeneration of the basal ganglia, along with hyperkinetic movement, seizures, cognitive impairment, and neuropsychiatric manifestations (Walker et al., 2011). Chorea-acanthocytosis (ChAc, OMIM 200150) occupies the main entity of this disease group which also includes McLeod syndrome (MLS), Huntington’s disease-like 2 (HDL2) and, more rarely, pantothenate kinase-associated neurodegeneration (PKAN) (Walker et al., 2011). From a perspective of genotype, ChAc mainly follows an autosomal recessive (AR) inheritance pattern (Danek et al., 2012), and the causative gene is vacuolar protein sorting 13A (*VPS13A*), a large gene consisting of 73 exons located in chromosome 9q21 (Walker et al., 2012). Up to now, the *VPS13A* gene mutation types reported ever include missense, nonsense, frameshift, duplication, deletion and splice site mutations (Velayos-Baeza et al., 2004). Chorein, the *VPS13A* gene expression product, is ubiquitously detected in a wide variety of human tissues (Dobson-Stone et al., 2004), which has been proven to be markedly reduced or absent in ChAc patients with the mutations described above (Dobson-Stone et al., 2004; Velayos-Baeza et al., 2004). While viewing from phenotypic aspects, ChAc can manifest as chorea, dystonia, cognitive impairment, seizures, psychosis, and even Parkinsonian features (Ueno et al., 2001), thus bearing immense resemblance to Huntington’s disease, dystonia, epilepsy, Parkinsonism, etc. Hence, the multifarious *VPS13A* mutation patterns, intricate symptom complex and overlapped clinical features enable diagnosis and differential diagnosis of ChAc an enormous challenge.

Herein, we report two clinical cases diagnosed with ChAc, varying from *VPS13A* mutation patterns, presenting manifestations, symptom spectrum to laboratory biomedical indicators. After a retrospective review of the ChAc case ever reported, we regrettably find that few cases are from China. Moreover, in light of the sharp contrast between population base and reported cases in China and the global prevalence of ChAc, it can be concluded that the morbidity of ChAc has been underdiagnosed in China (Liu et al., 2014). Based on the ChAc literatures ever published and these two cases, we conclude several suggestive features and propose a diagnostic path of ChAc, aiming to facilitate the future diagnosis and management of ChAc in China.

## Case Presentation

Two confirmed ChAc patients, once admitted into Department of Neurology, Union Hospital, Tongji Medical College, Huazhong University of Science and Technology (TJMC, HUST), were interviewed and surveyed retrospectively, and their medical records were thoroughly reviewed. Besides, relevant clinical and imaging data during the periodic outpatient follow-ups were consulted as well.

### Ethics Statement

This study was carried out in accordance with the guidelines of institutional ethics committee of Union Hospital, TJMC, HUST. All subjects gave written informed consent on the basis of the Declaration of Helsinki.

### Case 1

**FIGURE 1:**
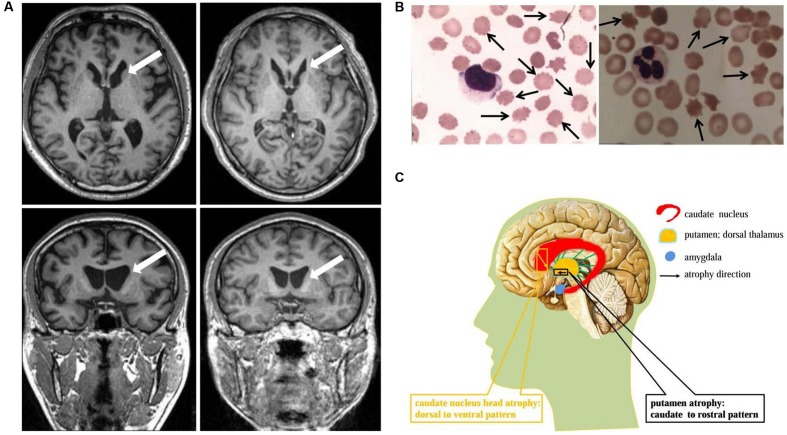
**Neuroimaging and cytological features identified in ChAc patients.** **(A)** Axial and coronal view of the basal ganglion plane in patient 1 (left) and patient 2 (right) demonstrated similar MR imaging features: caudate nucleus head atrophy and anterior horn dilation of lateral ventricles (marked by arrows). **(B)** Acanthocytes (marked by arrows, Giemsa staining) were detected in peripheral blood smear under light microscope examination. The average acanthocytes ratio in patient 1 and 2 were 18% (left) and 17% (right), respectively. **(C)** Schematic diagram of striatal atrophy pattern in ChAc patient. The caudate nucleus head atrophy demonstrated a dorsal to ventral pattern (marked by yellow), while the putamen presented a caudate to rostral mode (marked by black).

**FIGURE 2:**
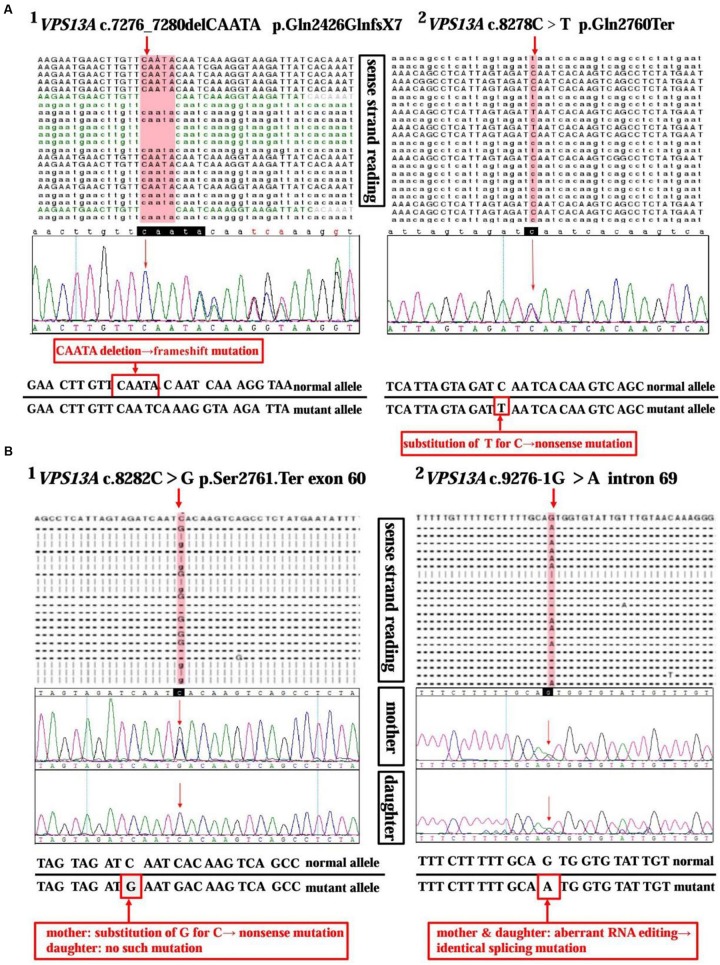
**Sequencing analysis of the *VPS13A* gene in ChAc patients and family member.** **(A)** High-throughput sequencing of *VPS13A* gene (NM_033305) in patient 1 reveals a 5 bp heterozygous deletion (c.7276_7280delCAATA) in exon 52 (A1) and a substitution of T for C (c.8278C>T) in exon 60 (A2). Moreover, the frameshift mutation and nonsense mutation have been confirmed by the Sanger sequencing method precisely. **(B)** Chip capture high throughput sequencing of *VPS13A* gene (NM_033305) in patient 2 shows a substitution of G for C (c.8282C>G) in exon 60 (B1) and an aberrant splicing pattern (c.9276-1G>A) in intron 69 (B2). While, in contrast, her daughter only shows a heterozygous splicing mutation similar to her mother’s in intron 69 (c.9276-1G>A) (B2), but not the nonsense one in exon 60 (B1).

A 37-year-old man, a warehouse keeper, presented with perioral chorea, dysphagia, dysarthria, vocalization, and involuntary upper limb movements for 5 months. His symptoms had been gradually progressing, aggravated recently by involuntary self-mutilation behaviors: tongue and lip biting. The past history revealed that he was diagnosed with generalized tonic–clonic seizure (GTCS) at age 30, but maintaining continuous remission with regular sodium valproate administration. On neurological examination, the patient showed perioral chorea, reduced muscle tone and tendon reflex and sporadic mouth ulcers, especially accompany frequent suck-mimicking activity. Upon admission screening stage, the laboratory biomedical tests revealed a considerable elevation in serum creatine kinase (CK), l-lactate dehydrogenase (LDH) and alpha hydroxybutyrate dehydrogenase (HBDH), especially for CK reaching up to seven-fold (see **Table 1**). The subsequent brain magnetic resonance imaging (MRI) scanning indicated moderate anterior horn dilation of lateral ventricles and mild caudate nucleus head atrophy (see **Figure 1A**-left column). Based on the clinical features, neurological examination findings and auxiliary test results available, we proposed the probable diagnosis of ChAc. Then four repeated and independent blood smears were performed, revealing that acanthocytes ratio averaged to 18% of the complete blood count (CBC) (see **Figure 1B**-left column). To further confirm the diagnosis, the *VPS13A* gene was sequenced subsequently. As expected, a heterozygous frameshift mutation in exon 52 (c.7276_7280delCAATA) and a heterozygous nonsense mutation in exon 60 (c.8278C>T) had been determined, which probably gave rise to the premature translation termination and even nonsense-mediated decay (NMD) pathway induced *VPS13A*-mRNA degradation (Kervestin and Jacobson, 2012) (see **Figure 2A** and **Table 1**). Besides, subsequent Sanger sequencing also confirmed the heterozygous frameshift and nonsense mutations (see **Figure 2A**). Moreover, the *HD-IT15* gene (CAG)_n_ analysis had also been performed to exclude the possible comrbidity of HD, but revealing no pathogenic mutations. In conclusion, the patient was ultimately diagnosed with ChAc and prescribed haloperidol, tiapride to control his perioral chorea (Diederich et al., 1990). Besides, given that vitamin E was once reported to relieve ChAc symptoms by means of improving red blood cell membrane fluidity (Hardie, 1989), it was tentatively administrated as well. As a result, his perioral chorea and self-mutilation behaviors were alleviated 3 days later and he was discharged after a week of clinical observation.

**Table 1:** A summary of the diagnostic evidences identified in the affected ChAc patients.

Items	Patient 1	Patient 2
Gender/age at onset	Male/37	Female/45
Occupation	Warehouse keeper	Housewife
Family history	–	–
Primary symptoms	Perioral chorea, frequent sucking–mimicking	Bruxism, involuntary tongue and lip biting
Incipient symptom	GTCS	Bruxism
Dystonia distribution	Oromandibular region, upper limbs	Oromandibular region
Dysarthria	+++	+
Tongue and lip biting	+	+++
Parkinsonian features	–	–
Tendon reflex	Areflexia of four limbs	Hyporeflexia of lower extremities
Seizure type	GTCS	–
Cognitive impairment	++	+
Psychiatric symptoms	Apathy, depression	Apathy, anxiety
EMG	Axonal neuropathy	Axonal neuropathy
CK (38-170U/L)	1122	137
LDH (109-245U/L)	314	175
HBDH (72-182U/L)	247	159
ALT (5-40U/L)	34	14
AST (8-40U/L)	47	15
Acanthocyte ratio of CBC	18%	17%
MRI	Moderate caudate nucleus head atrophy and paracele anterior horn dilation	Mild caudate nucleus head and putamen atrophy
*HD-IT15* gene (CAG)_n_ analysis	No pathogenic mutation	No pathogenic mutation
*VPS13A* sequence changes	c.7276_7280delCAATA c.8278C>T	c.8282C>G c.9276-1G>A
Chorein alterations	p.Gln2426GlnfsX7 p.Gln2760Ter	p.Ser2761Ter Splicing mutation
Mutation types	Frameshift mutation (exon52) Nonsense mutation (exon60)	Nonsense mutation (exon60) Splicing mutation (intron69)


*(+++, severe; ++, moderate; +, mild; –, none); GTCS, generalized tonic–clonic seizure; CK, creatine kinase; LDH, l-lactate dehydrogenase; HBDH, alpha hydroxybutyrate dehydrogenase; ALT, alanine transaminase/glutamic-pyruvic transaminase; AST, aspartate transaminase/glutamic–oxaloacetic transaminase; HD, Huntington’s disease; EMG, electromyography; CBC, complete blood count; Gln, glutamine; Ser, serine; Ter, termination codon.*

### Case 2

A 45-year-old housewife gradually developed involuntary bruxism (teeth grinding), dysphagia, dysarthria, vocalization, and frequent self-mutilation behaviors (tongue and lip biting) after a recovery from a severe mouth ulcer 1 month ago. Her orofacial chorea was characterized by clumsy, non-coordinated mandibular movement and occasional grimacing activities. A further family history inquiry revealed no similar symptom presenters. On neurological examination, she showed frequent teeth clenching, reduced limb muscle tone and tendon reflex and multiple lip ulcers. To prevent the risk of self-mutilation, she had to continuously hold a roll of cloth in her mouth. The serum CK, LDH, and HBDH levels are all within normal ranges (see **Table 1**). On the basis of clinical features and serum biomchemical indicators, the patient was temporarily diagnosed with oromandibular dystonia. But a routine blood smear test accidentally detected acanthocytes, which thereby led us to the assumptive diagnosis of ChAc. Subsequently, three repeated and independent peripheral blood smears conformably detected acanthocytes, with acanthocytes ratio averaged to 17% of CBC (see **Figure 1B**-right column). Besides, brain MRI scanning demonstrated mild atrophy in the caudate head and putamen bilaterally, accompanying moderate anterior horn dilation of lateral ventricles (see **Figure 1A**-right column). Moreover, the *VPS13A* gene sequencing further confirmed the diagnosis, revealing a heterozygous nonsense mutation (c.8282C>G→p.Ser2761Ter) in exon 60 and a heterozygous splicing mutation (c.9276-1G>A) in intron 69 (see **Figure 2B** and **Table 1**). Nevertheless, the *VPS13A* gene sequencing in this patient’s daughter, a healthy girl with no sign of ChAc, identified a heterozygous splicing mutation similar her mother’s in intron 69 (c.9276-1G>A) (see **Figure 2B**). As a matter of fact, the inheritance pattern of ChAc was reported to vary from AR inheritance (predominant pattern), autosomal dominant inheritance to X linked recessive inheritance pattern (Ueno et al., 2001; Dobson-Stone et al., 2002), accompanying different levels of penetrance in different sufferers or families (Saiki et al., 2003; Walker et al., 2007). Therefore, when postulating that ChAc is inherited in an AR inheritance and complete penetrance pattern in this family, the healthy girl is probably a carrier of pathogenic *VPS13A* gene. In conclusion, according to the clinical symptoms, neuroimaging features and, especially, genetic findings, the patient was finally diagnosed with ChAc. Then the patient received botulinum toxin (BTX) injection to relieve her torturous bruxism and self-mutilation behavior, eventually achieving prominent remission. In the outpatient follow-up 4 months later, she was also routinely prescribed haloperidol and vitamin E to control chorea (Diederich et al., 1990) and improve the red blood cell membrane fluidity (Hardie, 1989) and it subsequently presented satisfactory therapeutic effects.

## Discussion and Literature Review

In this study, we present two ChAc cases on the basis of clinical symptoms, neuroimaging features, and *VPS13A* genetic findings. While a retrospective study of the ChAc cases ever reported revealed that there probably exists a considerable underdiagnosis of ChAc in China (Liu et al., 2014). Given this stern status quo of ChAc management, we propose several suggestive features and a diagnostic path of ChAc (see **Table 2**), which can be used to facilitate the diagnosis and management of ChAc in China.

**Table 2:** Proposed diagnostic path of ChAc.

Evidence Categories	Details
I. Clinical clues (is mandatory)	① Hyperkinetic movements (e.g., perioral or oromandibular dystonia, feeding dystonia; grimacing activities, limb choreic movements) *most common cues*
	② Self-mutilation behaviors (especially tongue and lip biting; sporadic mouth ulcers) *most suggestive cues*
	③ Bruxism (teeth grinding behaviors)
	④ Dysarthria, dysphasia, articulation disorders
	⑤ Seizures attack (GTCS, simple or complex partial seizures)
	⑥ Parkinsonian symptoms (e.g., bradykinesia, rigidity)
	⑦ Neuropsychiatric symptoms (e.g., apathy, anxiety, depression, agitation, cognitive impairment)
	⑧ Diminished or absent tendon reflex (axonal neuropathy proved by EMG)
II. Neuroimaging traits	⑨ Caudatum (especially caudate nucleus head) and lenticula atrophy (determined by striatal volumetry and morphometry), anterior horn dilation of lateral ventricles
III. Laboratory inspection	⑩ Serum chemistry indicators: CK, LDH, HBDH, ALT, and AST elevation *CK elevation, comparatively more specific indicator*
	⑪ Acanthocyte detection (peripheral blood smear or scanning electron microscope inspection) *most suggestive evidence*
	⑫ Chorein detection (reduced or absent chorein blot) *most confirmatory evidence*
IV. *VPS13A* gene sequencing	⑬ Pathogenic *VPS13A* mutation (e.g. missense, nonsense, frameshift, duplication, deletion, and splice site mutations) *most confirmatory evidence*


*The diagnosis of ChAc is proposed to be established on the basis of four categorical evidences: clinical clues, neuroimaging traits, laboratory inspection, and *VPS13A* gene sequencing. According to different evidence combinations, the diagnosis of ChAc can be classified into three subcategories: possible ChAc, probable ChAc, and definite ChAc.*

*(1) Possible ChAc: ① ② + ⑨ or ⑩ are mandatory, optional items from ③ - ⑧ (excluding other organic diseases and dystonia);*

*(2) Probable ChAc: ① ⑪ are mandatory, optional items from ② - ⑬ (excluding other organic diseases and dystonia);*

*(3) Definite ChAc: ① + ⑫ or ⑬ are mandatory, optional items from ② - ⑪ (excluding other organic diseases and dystonia)*

*Attention: Apart from the mandatory items, the more supportive evidences, the more definite of the establishment of each diagnostic subcategory.*

### Clinical Features

Chorea-acanthocytosis mostly present in early adulthood, between age 20 and 40 and rarely before age 20 or after 50 (Walker et al., 2007), generally with hyperkinetic involuntary movements as typical symptoms (Walker, 2015). As a matter of fact, perioral chorea, bruxism and self-mutilation behaviors, demonstrated by the two patients, are all concrete manifestations of the hyperkinetic movements (see **Tables 1**, **2**). In particular, several previous reports have already indicated that self-mutilation, especially tongue and lip biting, were critically suggestive of ChAc (Walker et al., 2006, 2007; Gooneratne et al., 2014) (see **Tables 1**, **2**). What is interesting is that the self-mutilation behaviors can be considerably alleviated by holding a roll of cloth in mouth just as performed by patient 2. This phenomena can be interpreted as direct mechanic protections or a certain form of sensory trick (Bader et al., 2010; Walker, 2015).

Seizures and neuropathy are also relatively common features of ChAc (see **Tables 1**, **2**). It has been speculated that about 42% of patients have at least one seizure attack during their clinical courses (Rampoldi et al., 2002). In general, seizure are mostly antiepileptic drugs (AEDs) sensitive GTCS in ChAc, just as proven in patient 1, but simple and complex partial seizures have been described as well (Al-Asmi et al., 2005; Walker et al., 2007). For example, a recent study had identified a rare c.2343del *VPS13A* gene mutation in three Israeli families, in which most affected individuals presented seizures as the first and dominant symptom (Benninger et al., 2016). Moreover, seizures of this type could be of temporal lobe origin and were AEDs resistant in five out of nine patients (Benninger et al., 2016). Peripheral sensorimotor neuropathy with the absence of deep tendon reflexes is common in NA, and it can be no easy to differentiate from motor neuron disease (Neutel et al., 2012). Nevertheless, the neuroelectrophysiological abnormities predominantly prove to be axonal neuropathy in ChAc (Walker et al., 2007) (see **Tables 1**, **2**), which is quite different from that of motor neuron disease.

Dysphagia and dysarthria are also common symptoms in ChAc (see **Tables 1**, **2**), resulting from progressive swallowing and vocal musculature dysfunction. While in the initial stages swallowing and speech impairment are likely due to orolingual dystonia, patients often become mute as the disease proceeds into terminal stages, thus implying an involvement of central nervous system dysfunction (Walker, 2015). Additionally, Parkinsonism are also involved in the symptom spectrum as a presenting or accompanying symptom, especially in later stage with the encroachment of the direct and indirect pathways (Bostantjopoulou et al., 2000).

Patients diagnosed with ChAc often present neuropsychiatric symptoms prior to the development of movement disorders (Walker, 2015). In general, psychiatric symptoms include apathy, anxiety, depression, obsessive-compulsive disorders, and agitation, which are also precisely confirmed by the affected patients in this study (see **Tables 1**, **2**). Cognitive impairments range from mild cognitive impairment to dementia, with deficits mainly manifesting as memory decline and executive dysfunction.

Serum biochemistry reveals that CK, LDH, and HBDH are elevated in the vast majority (about 85%) of ChAc patients and it may precede the appearance of neurologic symptom as a sign of subclinical myopathy (Lossos et al., 2005; Walker, 2015). However, the serum biochemical alteration are not mandatory for the diagnosis of ChAc, just as proven by patient 2 whose CK, LDH, and HBDH values are all within normal ranges (see **Table 1**). Nevertheless, CK elevation is not associated with HDL2, PKAN, and HD, thus enabling serum CK a relatively more specific indicator for ChAc (see **Table 2**) especially in China when compared with other biochemical indicators (Liu et al., 2014).

Acanthocyte detection in peripheral blood smear is a specific indicator for the diagnosis of ChAc (see **Figure 1B** and **Table 1**). However, the detection efficiency of acanthocytes is largely dependent on several specific endogenous factors and blood sample treatment methods, and their absence does not absolutely exclude the diagnosis of ChAc (Bayreuther et al., 2010). Scanning electron microscopy is the most reliable morphological diagnostic method, but it is still not routinely popularized. Therefore, chorein detection (Velayos-Baeza et al., 2004) or *VPS13A* gene sequencing (Walker et al., 2012) can be proposed to be alternatives in the scenario of acanthocyte detection failure.

### Genetic Findings

ChAc, an AR inherited movement disorder, can be attributed to *VPS13A* gene mutation on chromosome 9q21. The previously reported *VPS13A* mutation patterns include missense, nonsense, frameshift, duplication, deletion, and splice site mutations (Dobson-Stone et al., 2004). In this study, the *VPS13A* gene sequencing in patient 1 revealed a heterozygous frameshift mutation in exon 52 (c.7276_7280delCAATA) and a heterozygous nonsense mutation in exon 60 (c.8278C>T), while in patient 2 and her daughter identified a heterozygous nonsense mutation (c.8282C>G) in exon 60 and a heterozygous splicing mutation (c.9276-1G>A) in intron 69 (see **Figure 2** and **Table 1**). In general, the mutations described above may probably give rise to premature translation termination, NMD pathway induced *VPS13A*-mRNA degradation (Kervestin and Jacobson, 2012), abnormal spliceosome formation and even production of aberrant chorein, which can genetically contribute to the occurrence of ChAc.

What is interesting is that a recent study revealed a rare c.2343del mutation in three ChAc Israeli families in which seizures present as the premier and prominent symptoms (Benninger et al., 2016). Moreover, the seizures presented in these families were, in large part, of temporal origin and partly AEDs resistant (Benninger et al., 2016). A retrospective review of ever published literatures indeed indicates the presence of epilepsy at early stages of NA (Hardie et al., 1991; Schwartz et al., 1992; Kazis et al., 1995; Aasly et al., 1999; Al-Asmi et al., 2005), but scarcely in the case of ChAc. In comparison, orofacial and oromandibular chorea are typical symptom in both affected patients in our study, but the presenting symptom in patient 1 is GTCS while in patient 2 is bruxism (see **Tables 1**, **2**). Given the novel and disparate *VPS13A* gene mutations identified in patient 1 and patient 2, it can be presumed that particular incipient symptoms may be associated with some specific *VPS13A* mutations, just as corroborated by the relevance of c.2343del mutation to epilepsy in Israeli families (Benninger et al., 2016). Nevertheless, restricted by the sample sizes in this study, it is imperative to investigate the intriguing proposition of genotype–phenotype correlation in larger ChAc groups in future study.

Hence, in light of the miscellaneous incipient symptom at early stages of ChAc, the *VPS13A* gene sequencing can be regarded as a reliable diagnostic and research tool. As a matter of fact, it is precisely the novel *VPS13A* gene mutations have finally helped to confirm the suspected ChAc cases in this study.

### Neuroimaging Traits

The ChAc patients, on postmortem, typically presented atrophy of the caudate nucleus, putamen, and globus pallidum, in some cases involving substantia nigra as well, but usually sparing cerebral cortex, cerebellum, locus coeruleus, inferior olives, and other brain regions (Hardie et al., 1991; Henkel et al., 2006). From a neuropathological perspective, marked neuronal loss and astrocytic gliosis were frequently observed, especially in the caudate nucleus (Ishida et al., 2009). Brain MR scanning in established ChAc patients also mirrored these findings, demonstrating similar macroscopic features, with caudate nucleus head being the most vulnerable region (Walterfang et al., 2008; Ishida et al., 2009) (see **Figures 1A,C**).

Given the significance of caudate nucleus in ChAc, several neuroimaging studies on caudate nucleus volume and shape alterations had been performed. By means of voxel-based morphometry (VBM) approach, Henkel et al. (2006) reported a focal and symmetrical atrophy in the caudate nucleus when comparing six patients with 15 age-matched controls. Later, Huppertz et al. (2008) further confirmed the conclusion with normalized VBM which could distinguish ChAc solely on the basis of caudate volumetry. These two studies had proven volume reduction in caudate nucleus and established a neuroimaging indicator (caudate volumetry) in the diagnosis of ChAc, but not investigating the morphometric changes. Walterfang et al. (2011) advanced the study with the help of non-parametric spherical harmonic technique which confirmed marked caudate shape alterations in ChAc. Nevertheless, the shape and volume alterations did not manifest uniformly, but with a prominent predilection to caudate nucleus head (Walterfang et al., 2011) (see **Figures 1A,C**). More specifically, the caudate nucleus atrophy revealed a dorsal to ventral gradient, while in putamen demonstrated a caudal to rostral pattern (Walterfang et al., 2011) (see **Figure 1C**). This particular pathological changes might be associated with the neuropsychiatric or cognitive symptoms in the early stage of ChAc, in light of the crucial role of caudate nucleus in frontostriatal loops (Leh et al., 2007; Draganski et al., 2008; Utter and Basso, 2008). In conclusion, based on the neuroimaging features identified in ChAc, the striatal volumetry and morphometry could be proposed as promising neuroimaging diagnostic indicators in future clinical practice.

## Conclusion

Here in this study, we present the clinical symptoms, neuroimaging features and *VPS13A* gene sequencing results of two patients, which all finally support the diagnosis of ChAc. Besides, four novel *VPS13A* gene mutations have been identified, which genetically corroborate the pathogenesis of ChAc and expand the *VPS13A* gene mutation spectrum as well. Nevertheless, it has been revealed that only a handful of cases are from China in a retrospective review of the published ChAc cases (Liu et al., 2014). Hence, in light of China’s tremendous population base, rare ChAc case reports and the overall prevalence worldwide, it can be presumed that there may exist an underdiagnosis of ChAc in China (Liu et al., 2014).

Confronted with such frustrating status quo, we have concluded several suggestive indicators and proposed a diagnostic path of ChAc on the basis of ever published reports and the present two cases, which falls into the range of clinical clues, neuroimaging traits, laboratory inspections and genetic findings, respectively: (1) clinical clues: oromandibular and feeding dystonia, self-mutilation behaviors (tongue and lip biting); (2) neuroimaging traits: paracele anterior horn dilation and caudate nucleus head atrophy; (3) laboratory inspections: serum CK elevation and acanthocytes detection; (4) genetic findings: familial aggregation, positive family history, and pathogenic *VPS13A* gene mutations discovery; (see **Tables 1**, **2**). In conclusion, we expect the reported cases and proposed diagnostic path could facilitate the future diagnosis and management of ChAc in China.

## Author Contributions

YS, NX, and XLi conceive and draft of the manuscript; YS, XLo, CH, FW, WF, XG, KM, SG, LW, YX, and LL equally contribute to the collection and sorting of the data and documents; ZL, NX, JH, and YS manipulate the polishing of the language and writing style; TW manages the integral envisage and top-layer design of the manuscript.

## Conflict of Interest Statement

The authors declare that the research was conducted in the absence of any commercial or financial relationships that could be construed as a potential conflict of interest.
